# BDNF Val66Met Polymorphism: Suggested Genetic Involvement in Some Children with Learning Disorder

**DOI:** 10.1007/s12031-022-02095-7

**Published:** 2022-12-23

**Authors:** Mohamed E. Elhadidy, Ayman Kilany, Ola Hosny Gebril, Neveen Hassan Nashaat, Hala M. Zeidan, Amal Elsaied, Adel F. Hashish, Ehab Ragaa Abdelraouf

**Affiliations:** 1 Children with Special Needs Research Department, Medical Research and Clinical Studies Institute, National Research Centre, Cairo, Egypt; 2 Pediatric Neurology Research Field, Medical Research Centre of Excellence, National Research Centre, Cairo, Egypt; 3 Learning Disability and Neurorehabilitation Research Field, Medical Research Centre of Excellence, National Research Centre, Cairo, Egypt

**Keywords:** BDNF, Val66Met gene, Polymorphism, Children, Learning disorder, Dyslexia

## Abstract

Brain-derived neurotrophic factor (BDNF) plays an essential role in neuronal survival, especially in areas responsible for memory and learning. The BDNF Val66Met polymorphism has been described as a cognitive modifier in people with neuropsychiatric disorders. BDNF levels have been found to be low in children with learning disorder (LD). However, Val66Met polymorphism has not been studied before in such children. The aim was to investigate the presence of BDNF val66Met polymorphism in a group of children with specific LD and to verify its impact on their cognitive abilities. The participants in this cross-sectional study (*N* = 111) were divided into two groups: one for children with LD and the other for neurotypical (NT) ones. Children with LD (*N* = 72) were diagnosed according to the DSM-5 criteria. Their abilities were evaluated using Stanford–Binet Intelligence Scale, dyslexia assessment test, Illinois Test of Psycholinguistic Abilities, and phonological awareness test. Genotyping of BDNF Val66Met polymorphism was performed for all participants. The frequency of the Met allele was 26% among children with LD (6 children had homozygous, 26 had heterozygous genotype). The percentage of participants with deficits in reading, writing, and phonemic segmentation was higher in Met allele carriers when compared to non-Met allele carriers in LD group. The frequency of Met allele among NT children was 3.85% (0 homozygous, 3 children had heterozygous genotype) (*p* = 0.00001). The high frequency of Val66Met polymorphism among children with LD introduces the BDNF gene as a genetic modifier of learning performance in some children who manifest specific learning disorder (developmental dyslexia).

## Introduction

Brain-derived neurotrophic factor (BDNF) is the most abundant neurotrophin expressed in the central nervous system and was reported to be involved in neuronal growth and differentiation and synaptic plasticity, especially in the frontostriatal–cerebellar circuits and ventral striatal–limbic circuits (Faris et al. 2020). These circuits are critical for the development of memory and visual motor integration abilities. The proper quality of connections between these areas is an essential prerequisite for the process of learning to read and write (Baroni and Castellanos 2015). BDNF levels are minimal during prenatal development but significantly higher postnatally, indicating its importance for neurogenesis as the brain matures (Karege et al. 2002). Patients suffering from psychiatric and neurodegenerative disorders such as major depressive disorder and Parkinson’s disease were reported to have low BDNF concentrations in their brains and blood. These disorders were found to be associated with memory deficits and a reduction of some cognitive abilities (Lima Giacobbo et al. 2019).

The human BDNF gene is localized on chromosome 11p14.1. It encodes a 247-amino acid preprotein which is, later on, proteolytically cleaved to form a 120-amino acid mature protein (Palasz et al. 2020). The BDNF gene consists of five alternatively used 5′ exons and a major 3′ exon. The alternative splicing of these 5′ exons would produce six different transcripts. This process leads to three isoforms of the preproprotein (a, b, and c) which differ in the length of their signal peptide and the brain areas or even cells parts (dendrites or soma) in which they are expressed (Martinowich and Lu 2008).

A single-nucleotide polymorphism (SNP) at the pro-region of BDNF (rs6265) is termed the Val66Met polymorphism (G196A). In this polymorphism, a valine (Val) to methionine (Met) change at position (codon) 66 of the proBDNF protein occurs. It is a sort of missense mutation which results in alteration of the coding strand of the gene which is responsible for a certain amino acid. Missense mutations do not necessarily lead to an observable change in the function of the produced protein. Sometimes, the resulting protein fails to function properly or becomes unstable and rapidly degraded. The produced amino acid or protein could fail to localize to its proper intracellular position. This polymorphism leads to deficient translocation and activity-dependent secretion of mature BDNF (Tsai 2018). It has been linked to impaired cognitive functioning in healthy adults and to several clinical features of many neuropsychiatric disorders such as major depressive disorder, Parkinson’s disease, and Alzheimer’s disease, in addition to diabetic patients (Bakusic et al. 2021; Cagni et al. 2017; Faris et al. 2020; Miranda et al. 2019). Performance of memory was one of the first cognitive domains to be extensively investigated for the influence of Val66Met polymorphism. Different types of memory impairment have been associated with the 66Met allele mutation in mice and human studies (Dincheva et al. 2012; Spencer et al. 2010). Long-term memory and working memory have been found to be particularly affected by restricted activity-dependent secretion of BDNF (Bakusic et al. 2021). Children with specific learning disorder (LD) were reported to manifest working memory deficits. Despite not having intellectual disability, such children fail to learn reading, writing, and spelling, and they usually manifest phonological awareness problems. Furthermore, children with LD were reported to have lower BDNF blood levels when compared to neurotypical (NT) children (Elhadidy et al. 2019).

Although the BDNF gene Val66Met polymorphism was a commonly studied gene variant within the field of cognitive neuroscience (Dincheva et al. 2012), it has not been studied in children with specific learning disorder before. Therefore, the aim of this study was to investigate the presence of the BDNF gene Val66Met polymorphism in a sample of Egyptian children with specific learning disorder (developmental dyslexia) compared to a sample of NT children. This would explore its possible relation to the learning abilities and cognitive performance of such children.

## Methods

A cross-sectional study which fulfilled the STROBE checklist was carried out. The participants with specific learning disorder (developmental dyslexia) were recruited from the learning disability and neurorehabilitation research clinic and the pediatric neurology research clinic (Medical Research Centre of Excellence, National Research Centre, Cairo, Egypt). They visited these clinics in the year 2021, complaining of poor scholastic achievement. Their age range was between 6.5 and 12 years (*N* = 72; 42 males, 30 females; IQ, 90–112, 96.5 ± 5.6). They were diagnosed in accordance with the criteria of the fifth edition of the diagnostic and statistical manual of mental disorders (American Psychiatric Association 2013). The control subjects (NT children) were friends of the LD participants (*N* = 39; 21 males, 18 females). Their parents agreed to join the study and they were age, gender, and socioeconomic status matched to the first group. All participants were enrolled in the national education system and got proper education opportunities and, therefore, were included. Children who manifested neurological examination abnormalities, 1 h EEG abnormalities, associated neurodevelopmental disorders, sensory deficits, intellectual disability, or a history of motor developmental delay were excluded from the study. The study was approved by the medical research ethics committee of the National Research Centre. The child’s assent and written informed consents were taken from the parents of participants.

The intellectual, reading, and other related cognitive abilities of children with LD were assessed by the following:

- The 4th edition of the Sanford–Binet Intelligence Scale (Melika 1998; Thorndike et al. 1986).
- The dyslexia assessment test which has 11 subtests. The raw scores of the subtests were used to obtain scaled scores for each subtest using standardized tables for certain age groups. They were also used to obtain an “at-risk” quotient. The scaled scores are in the form of grades (-,--,---, 0, +) where the first 3 scores represent deficits and the scores 0 and + represent good and superior performance (Aboras et al. 2008; Fawcett and Nicolson 1996).
- The Illinois Test of Psycholinguistic Abilities which has 10 subtests. The raw scores obtained in each subtest were converted into scaled scores according to standardized tables for each age group. The deficits in the subtests were determined when the scaled score in a certain subtest was more than 6 scores below the mean of the sum of the scaled scores of the subtests. For example, when the sum of scaled scores for a child was 360, the mean of his scaled scores is 36, and obtaining a scaled score less than 30 in a certain subtest indicated a deficit in this subtest (El-Sady et al. 1996; Kirk et al. 1986).
- The phonological awareness test was used to evaluate the word, syllable, rhyme, and phoneme awareness. Deficits were considered present when the total score of the test for a child was less than the 5th percentile values of the test (El-Sady et al. 2011).

### Genotyping Analysis

- Genomic DNA was extracted from whole blood using QIAamp DNA Blood Mini Kit (Cat. No. 51104, for Molecular Biology Applications, Product of Germany).
- BDNF Val66Met genotyping was performed as per the laboratory’s standard protocol by utilizing the following primers for G196A in the BDNF gene: forward 5-ATC CGA GGA CAA GGT GGC-3 and reverse 5-CCT CAT GGA CAT GTT TGC AG-3. This generated 300 bp of polymerase chain reaction (PCR) products, which were subsequently digested by Pml1 to yield either allele A (met: undigested, 300 bp) or allele G (val: digested to 180 bp + 120 bp bands). They were visualized on an agarose gel by using a 50 bp marker (Shimizu et al. 2004). Participants were classified as either BDNF Met allele carriers (Val/Met or Met/Met genotype) or BDNF non-Met allele carriers (Val/Val genotype). The percentages of the detected genotypes, either Val/Met (heterozygous genotype), Met/Met (homozygous genotype), or Val/Val (wild genotype), were calculated according to the frequency of those genotypes in relation to the total number of alleles in each group, not in relation to the number of participants (144 alleles in the LD group; 78 alleles in the NT children group).

The clinical and genetic tests were performed and rechecked by different examiners or under the supervision of another examiner to avoid bias.

### Statistical Analysis

For data analysis, IBM SPSS version 22.0 (IBM Corp., Chicago, USA, 2013) was used. Quantitative data were represented as mean ± SD (standard deviation), and inferential analysis was performed using the independent *t* test. Number and percentage were used for qualitative variables, and the chi-square test was used for the analysis. Regarding the dyslexia assessment test, the scaled scores were converted into a scoring qualitative system to be suitable for statistical analysis. Grades were given to the scaled scores to be suitable for identifying the deficits in the subtests. Grades 1, 2, and 3 represented deficits in the performance of the measured abilities (1 represented the weakest performance), while grades 4 and 5 represented good and superior functioning. The percentages of participants who manifested deficits in the subtests of the dyslexia assessment test and the Illinois Test of Psycholinguistic Abilities among the LD group were used for comparison between the Met allele and the non-Met allele carriers among children with LD. The percentages of participants who manifested deficits in the total score of the phonological awareness test were used for comparison among children with LD. The level of significance was taken when *p* value was < 0.05.

## Results

### BDNF Genotyping Results (Val66Met)

In the group of children with LD, the frequency of the Val allele (non-Met allele) was 74%, while the frequency of the Met allele was 26% among all examined alleles of children with LD. The number of cases with wild genotype was 40; the number of children with the heterozygous genotype was 26, while 6 children had the homozygous genotype.

**Fig. 1:**
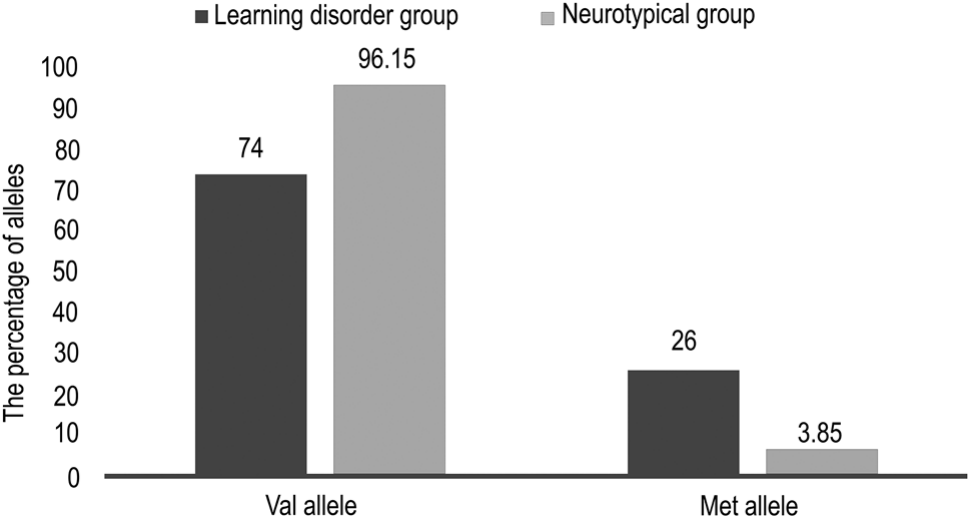
The percentage of Val allele and Met allele in the learning disorder group versus the neurotypical group

**Fig. 2:**
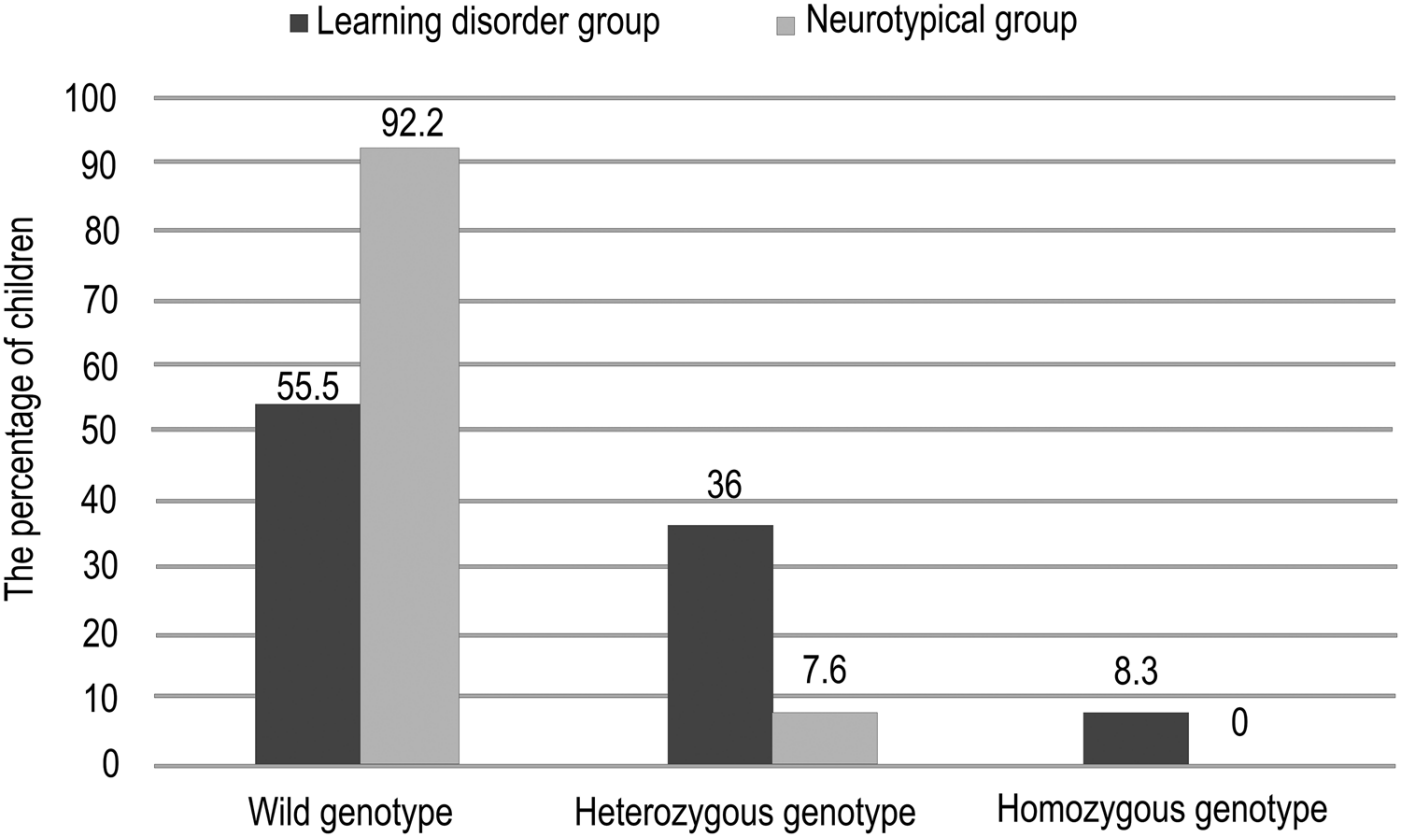
The percentage of children who have wild, heterozygous, or homozygous genotypes in the learning disorder group versus the neurotypical group

In the control group of NT children, the frequency of Val allele was 96.15%, while the frequency of Met allele was 3.85%. There were 36 wild children and 3 heterozygous children (Figs. 1 and 2). There was a significant statistical difference between the group of children with LD and the NT children regarding the frequency of the Met allele (*p* value = 0.000013).

### Results of the Used Tests to Assess the Abilities of Children with Learning Disorder

The at-risk quotient scores obtained by the dyslexia assessment test were 1 or more (range, 1–2.6; mean, 1.7 ± 0.5). Therefore, all children in LD group manifested deficits in reading and other related cognitive abilities. The results of the scaled scores of the subtests of the Illinois Test of Psycholinguistic Abilities indicated that some of these children manifested deficits in all subtests except verbal expression and manual expression. The percentages of participants with deficits were high in auditory reception, auditory association, auditory sequential memory, and visual closure. The raw scores of the phonological awareness test ranged from 38.5 to 110 (72.04 ± 23.7). The percentage of participants who had deficits in the phonological awareness test was 77.7%

### Comparison Between Met Allele Carriers and Non-Met Allele Carriers Regarding the Used Tests in Children with LD

**Fig. 3:**
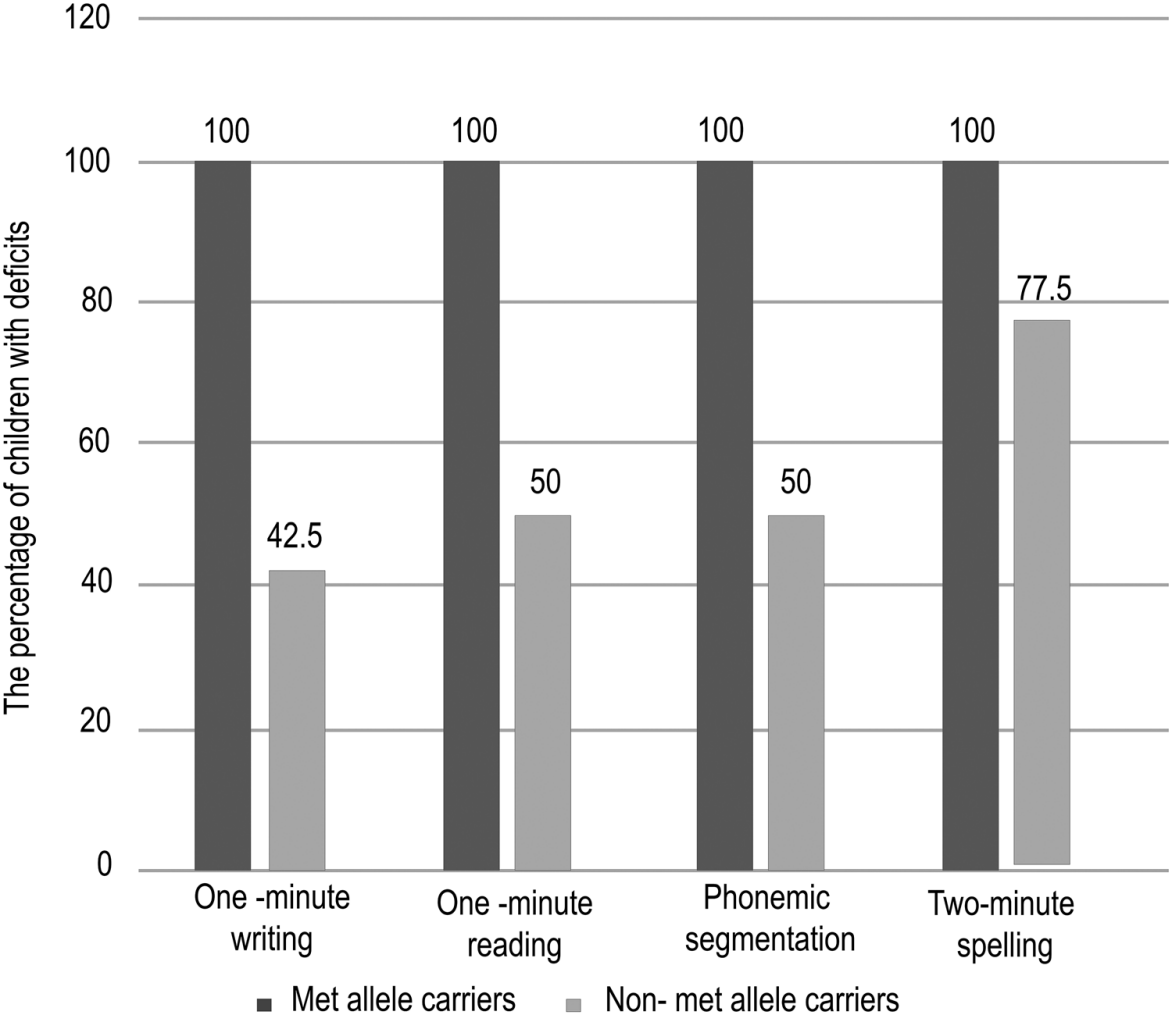
Comparison between Met allele carriers and non-Met allele carriers in children with learning disorder regarding the percentage of participants with deficits in some subtests of the dyslexia assessment test

The IQ scores of the Met allele carriers (95.7 ± 8.9) were lower compared to those of the non-Met allele carriers (96.4 ± 6.5). Yet, no significant statistical difference was noticed (*p* = 0.7). It has been noticed that the percentages of participants who manifested deficits in 1-min reading, nonsense passage reading, 1-min writing, 2-min spelling, posture stability, phonemic segmentation, backward digit span, rapid naming, and verbal fluency subtests of the dyslexia assessment test were higher in the Met allele carriers (*N* = 32) when compared to the non-Met allele carriers (*N* = 40). In other words, the deficits in many of the evaluated abilities were more prevalent among the Met allele carriers compared to non-Met allele ones. The bead threading and semantic fluency did not show such a pattern. A significant statistical difference was noticed only regarding 1-min writing, 1-min reading, phonemic segmentation, and 2-min spelling (*p* < 0.0001, < 0.0001, < 0.0001, *p* = 0.009, respectively), and these abilities were represented in Fig. 3. Furthermore, the percentages of the participants who manifested deficits in most of the subtests of the Illinois Test of Psycholinguistic Abilities were higher in the Met allele carriers (Val/Met or Met/Met genotype) compared to non-Met allele carriers. Only the percentages concerning auditory association, auditory sequential memory, visual sequential memory, visual closure, and grammatic closure showed significant statistical difference (*p* = 0.04, 0.007, 0.01, < 0.0001, 0.02). None of the participants in the LD group manifested deficits in the verbal and manual expression subtests. Regarding the phonological awareness test, no significant statistical difference was noticed when comparing the percentage of participants with deficits in the met allele and non-Met allele carriers (Table 1).

**Table 1:** Comparison between Met allele carriers and non-Met allele carriers in children with learning disorder regarding the percentage of participants who manifested deficits in some subtests of the Illinois Test of Psycholinguistic Abilities and the phonological awareness test

Items	Percentage in Met allele carriers (%)	Percentage in non-Met allele carriers (%)	*p* value
Auditory reception	65.6	52.5	0.2
Auditory association	53.1	30	0.04*
Auditory sequential memory	56.2	25	0.007*
Visual reception	31.25	22.5	0.4
Visual association	18.75	7.5	0.1
Visual sequential memory	15.6	0	0.01*
Visual closure	84.3	32.5	< 0.0001*
Grammatic closure	12.5	0	0.02*
Phonological awareness test	87.5	70	0.3

*Significant

## Discussion

Specific learning disorder (LD) with impairment in reading or developmental dyslexia is a very mysterious developmental disorder with several proposed etiological factors. Many alterations have been accused of sharing in the pathogenesis of this disorder, such as altered brain myelination and functioning of different brain areas involved in reading, memory, and language processing, such as temporoparietal region, internal capsule, corpus callosum, hippocampus, and thalamus (Beaulieu et al. 2020; Feldman et al. 2010). Altered connections between different brain areas involved in learning were also reported (El-Sady et al. 2020). Therefore, neurotrophic factors such as BDNF which modulate brain growth, maturation, and function are suspected to have a role in the brain alterations detected in individuals with specific LD.

Children are diagnosed with developmental dyslexia or specific learning disorder when their intellectual abilities are within normal ranges, yet they fail to acquire the proper levels of reading, spelling, and writing words or sentences dictated to them (American Psychiatric Association 2013; Shaywitz 1998). Several theories explained the occurrence of reading disorder despite normal intelligence. Phonological theory is one of them, which emphasizes the role of phonological awareness deficits in the etiology dyslexia (El-Sady et al. 2011). Some visual and auditory processing abnormalities have been also proposed to be involved in reading disorder (Roach and Hogben 2007; Törmänen and Takala 2009). The results of the IQ assessment in this study indicated that the intellectual performance of participants with LD was within the normal range. However, the reading, writing, and other abilities involved in learning were found to be defective in the majority of participants. This is in agreement with previous reports investigating some cognitive abilities involved in learning, such as Elwan et al. (2019) and Nashaat et al. (2017), who reported the presence of deficits in some cognitive abilities in children with developmental dyslexia despite having normal ranges of intelligence quotients. Such deficits could be related to different brain activation and brain connectivity in areas involved in reading and phonological awareness tasks, such as temporoparietal and occipitotemporal areas. Furthermore, the right hemisphere tracts connectivity measures were related to the reading performance of children with dyslexia which differed from neurotypical children who rely on left hemisphere. Individuals with dyslexia used more areas in the brain when performing reading tasks compared to controls. These specific alterations could explain the reading deficits despite the normal intelligence (El-Sady et al. 2020; Prasad et al. 2020).

This is the first study that targeted the Val66Met polymorphism in children with specific LD. The percentage of the Val66Met polymorphism was 26% among children with LD. None of the children in the control group showed the homozygous Met allele. Furthermore, the defective reading, writing, spelling, and phonemic segmentation abilities together with some other cognitive abilities were more prevalent among the BDNF gene Met allele carriers compared to non-Met allele carriers in the LD group. Such findings highlight the potential role of the BDNF Val66Met polymorphism in interfering with the abilities and performance of children with learning disorder. These outputs are in line with Dincheva et al. (2012) and Xie et al. (2020) who reported that the Val66Met polymorphism modulates aspects of cognitive functions even in healthy adults. Moreover, genetic association studies have stated that this polymorphism could be a possible risk factor for the development of neuropsychiatric disorders and their associated cognitive and memory impairments (Miranda et al. 2019; Chen et al. 2017). Another study conducted by Jasińska et al. (2017) investigated such polymorphism in healthy children and reported that Met allele carriers exhibited larger hippocampal volumes which underscores the influence of this polymorphism on the brain. Nevertheless, no influence has been noticed on verbal or performance IQ of such children (Jasińska et al. 2017). It is worth noting that the percentage of the Met allele in NT children in this study was lower than the percentages reported in the general healthy population in other countries like Italy and Romania, which were 17% and 19%, respectively (Liguori et al. 2009; Vulturar et al. 2016). Nevertheless, the frequencies in these studies were even lower compared to the frequency noticed among children with LD in this study. Furthermore, the intellectual abilities of these children were within normal despite having deficits in learning. This could reflect the burden of the Met allele on the abilities of those children and highlights the genetic role of this polymorphism in the included children with LD. The number of participants in this study was relatively small, which could be considered a limitation. Furthermore, generalizability cannot be applied. Nonetheless, this is the first study that investigated such polymorphism in children with specific LD or developmental dyslexia and the first study to spotlight the possible involvement of this genotype in LD.

The G196A polymorphism modifies intracellular packaging, distribution, and release of BDNF protein. It has a significant negative impact on episodic verbal memory and hippocampal activation, in addition to hippocampal neuronal integrity and synaptic abundance represented by lower hippocampal volume and N-acetyl aspartate levels in individuals with this mutation when compared to controls (Egan et al. 2003; Frodl et al. 2007; Hariri et al. 2003). This polymorphism is located on the 5′BDNF precursor peptide sequence (proBDNF), which is proteotically cleaved to form the mature form of the protein post-translationally. This process is achieved by tissue plasminogen activator enzyme, which was also implicated in psychiatric disorders (Rauti et al. 2020).

There are different possible routes for how this BDNF gene polymorphism could influence the functioning of different brain areas involved in learning. BDNF is a vital neurotrophin which is expressed in the brain throughout life. It serves as an essential neurotransmitter modulator and participates in mechanisms of neuronal plasticity such as long-term potentiation and learning. It is an established regulator of dendritic spine density and other molecular correlates of synaptic functioning, which is believed to underpin cognitive performance (Rauti et al. 2020). BDNF enhances the rise in the number of dendrites on neurons and neurotransmitter receptors in synapses, which then facilitates connectivity in the brain. The formation of additional receptors and neurons leads to more efficient learning and memory capacity and improves overall brain function. This action could be established through binding TrkB receptors, while the opposite can happen by activating the p75 receptor to which proBDNF usually binds. Low midbrain BDNF activity was implicated in striatal dopaminergic dysfunction (Razgado-Hernandez et al. 2015). BDNF was also implicated in the proper functioning of glutamate, GABA, and serotonin systems in different brain areas, including pyramidal and hippocampal neurons. On the postsynaptic side, BDNF augments the opening of NMDA receptor channels and upregulates the expression of Ca ++ –Na + channels at the cell membrane (Rauti et al. 2020).

Children enrolled in schools and not performing well are exposed to stress by their parents and teachers (Panicker and Chelliah 2016). Such stress could have contributed to exaggerating the hazards of the detected polymorphism, considering that BDNF expression is influenced by stress. Carballedo et al. (2013) stated that Met allele carriers who were exposed to childhood trauma (early life stress) showed lower hippocampal volumes as compared to carriers without early life stress. Therefore, reducing stress exposure and measures to overcome the BDNF malfunctioning should be emphasized during management and rehabilitation of such children. Consequently, the relationship between BDNF gene polymorphism and the development of abilities related to memory and learning to read and write is multifactorial and complicated and has different mechanisms. These mechanisms interlace together to negatively impact the performance of children with specific LD and contribute to the modulation of their adaptive learning behavior.

## Conclusion

The BDNF gene is a contributor to brain functions. Some abilities of reading, writing, spelling, and phonological awareness of the Met allele carriers in the learning disorder group were influenced by this genotype. The Val66Met polymorphism of the BDNF gene could be proposed as a genetic modifier of learning performance in some children who manifest specific learning disorder with impairment in reading or developmental dyslexia.

## Data Availability

The datasets used and/or analyzed during the current study are available from the corresponding author on reasonable request.
